# From the archives: Circadian clock regulation of phyA, regulation of Z-ring positioning in chloroplast division, and ADP–glucose pyrophosphorylase subunit interactions

**DOI:** 10.1093/plcell/koad144

**Published:** 2023-05-18

**Authors:** Arpita Yadav

**Affiliations:** Assistant Features Editor, The Plant Cell, American Society of Plant Biologists, USA; Biology Department, University of Massachusetts, Amherst, MA 01003, USA

## July 2022: Circadian clock regulation of phyA

Light signals are crucial for plant growth and development. Phytochromes are photoreceptors that respond to variations in light intensity and quality to set off a chain reaction that controls plant growth and development. Work by Wang et al. (2022) investigated phytochrome A (phyA) involvement in circadian clock control of hypocotyl development in *Arabidopsis thaliana*. Using dawn-phased signaling, the authors identified TIME FOR COFFEE (TIC) as a component of the circadian clock that controls phyA-mediated hypocotyl development. They showed that TIC has a light-dependent interaction with phyA, which is most potent at night’s end and dawn, when the circadian clock is in its most active state. They further demonstrated that the regulation of hypocotyl growth is facilitated by a connection between TIC and PIF4, a transcription factor that acts as a downstream effector of phyA signaling.The authors proposed a model in which TIC functions as a dawn-phased signaling component that strengthens the connection between phyA and PIF4 and stimulates the expression of phyA target genes, ultimately resulting in the regulation of hypocotyl development in response to light. Overall, this research shed light on how the circadian clock controls plant growth and development in response to light cues (Fig.).

**Figure.**
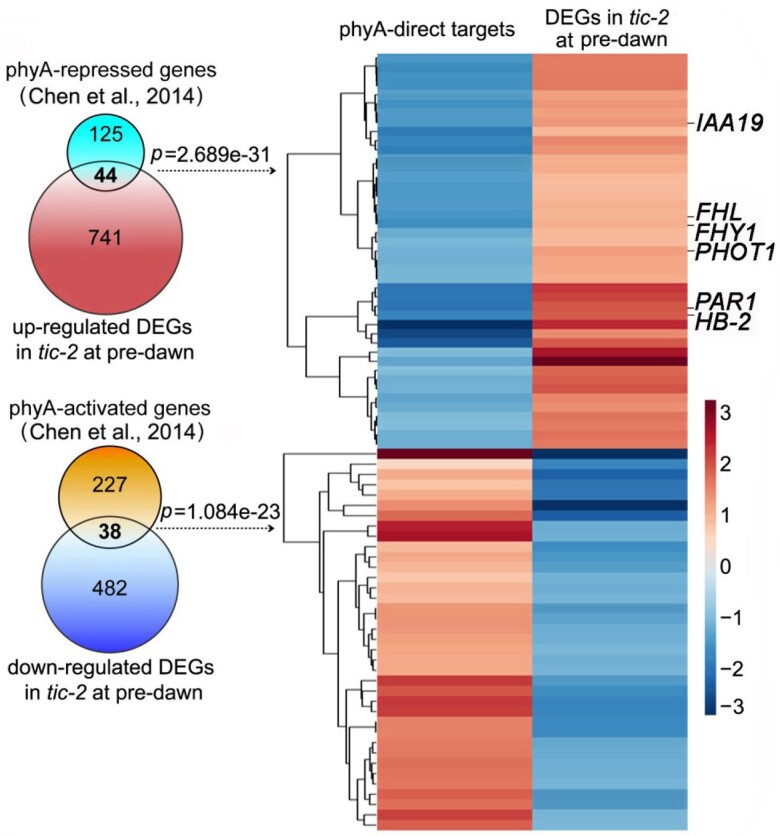
TIC and phyA regulate the transcription of a subset of genes in an opposite manner. Venn diagram (left) showing the number of overlapping genes between phyA direct targets (identified by Chen et al. 2014) and DEGs in *tic-2* at pre-dawn and a heatmap (right) showing the hierarchical clustering of the target genes that were co-regulated by phyA and TIC. Scale represents fold change. Reprinted from Wang et al. (2022) Figure 2c.

## August 2018: regulation of Z-ring positioning in chloroplast division

At the site of photosynthesis, chloroplasts play a crucial role in a plant’s ability to live, grow, and develop, and their regular division is necessary for cellular homeostasis. Multiple proteins and chemical processes are involved in chloroplast division, although the precise mechanisms involved remain unknown. Chen et al. (2018) investigated 2 proteins, MCD1 and ARC6, for their potential roles in chloroplast division in *Arabidopsis thaliana*. Conserved protein MCD1 is required for efficient chloroplast division, and the membrane-tethering protein ARC6 plays a role in chloroplast positioning. Using a variety of biochemical, genetic, and imaging techniques, the authors discovered that ARC6 is responsible for the connection of MCD1 with the chloroplast division machinery known as FtsZ filaments. The authors demonstrate that MCD1 and ARC6 are essential for the correct placement of FtsZ filaments and the establishment of the chloroplast division site. Furthermore, they show that MCD1 is necessary for FtsZ filaments to assemble into a ring-like structure at the division site. The authors suggest a model in which MCD1 acts as a scaffold protein that binds to FtsZ filaments at the chloroplast division site via ARC6 and directs their positioning and assembly. Thus they proposed that MCD1 controls FtsZ filament dynamics during chloroplast division.

## August 1998: ADP–glucose pyrophosphorylase subunit interactions

The endosperm of maize kernels is an important starch storage tissue because it supplies energy for germination and early growth of the seedling. ADP-glucose pyrophosphorylase (AGPase) is a complex enzyme with 2 types of subunits that catalyzes starch production. The Shrunken2 (Sh2) gene encodes the large subunit responsible for catalytic activity, and the Brittle2 (Bt2) gene encodes the small subunits that act as regulators of enzyme activity. Greene and Hannah (1998) investigated how the Sh2 and Bt2 AGPase subunits interact with one another in the endosperm of maize. They demonstrated that alterations to the Bt2 subunit cause aberrant endosperm growth, decreased AGPase activity, and changes to starch production. To determine the structural and functional roles of the Bt2 subunit, they used a combination of x-ray crystallography, biochemical, and genetic techniques and discovered a crucial interaction between the Bt2 and Sh2 subunits. The work showed that Bt2 helps to maintain the structure of the Sh2 subunit, which in turn increases its catalytic activity.
